# PROBLEMS AND PROSPECTS OF TECHNOLOGICAL AND BIOPHYSIOLOGICAL STUDIES OF TECHNOSTRESS IN MID-LIFE ADULTS

**DOI:** 10.1093/geroni/igz038.3452

**Published:** 2019-11-08

**Authors:** Elizabeth Mack, Shelia Cotten, Chu-Hsiang Chang, Wenda Bauschpies

**Affiliations:** Michigan State University, East Lansing, Michigan, United States

## Abstract

Long-term exposure to stress places people at risk for chronic diseases including but not limited to obesity, Type-2 diabetes, and heart disease. Various aspects of technology use are associated with stress. Known as technostress, this unique stress is characterized by individuals’ inability to cope with demands generated by computer-related technologies. To date, studies on technostress have focused on young adults and older adults, with an emphasis on self-reported indicators of both technology use and stress. This study differs from prior work in two ways. One, it examines technology use and stress in mid-life adults (50-64), an understudied population in research on technostress. This segment of the population is important because their technostress may negatively affect their successful transition into older adulthood. Second, we use three types of data to elucidate the linkages between technology use and stress: (1) self-reported survey measures of technology use and stress; (2) objective measures of technology use from tracking applications, and (3) biophysiological measures of stress. The study focuses on smartphone use, which was the most commonly used technology by mid-life adults on both weekdays and weekends based on our initial results (N=40). The goal of this pilot study is to highlight the problems and prospects of conducting technostress research through the utilization of multiple data collection modes: self-report, tracking applications (apps), and biophysical indicators.

